# Holistic care program for elderly patients to integrate spiritual needs, social activity, and self-care into disease management in primary care (HoPES3): study protocol for a cluster-randomized trial

**DOI:** 10.1186/s13063-019-3435-z

**Published:** 2019-06-18

**Authors:** Cornelia Straßner, Eckhard Frick, Gabriele Stotz-Ingenlath, Nicola Buhlinger-Göpfarth, Joachim Szecsenyi, Johannes Krisam, Friederike Schalhorn, Jan Valentini, Regina Stolz, Stefanie Joos

**Affiliations:** 1 Department of General Practice and Health Services Research, University Hospital Heidelberg, Im Neuenheimer Feld 130.3, 69120 Heidelberg, Germany; 2 Department of Psychosomatic Medicine and Psychotherapy, Research Center Spiritual Care, Technical University of Munich, Langerstr. 3, 81675 München, Germany; 3 Department for Medical Biometry, Institute for Medical Biometry and Informatics, University Hospital Heidelberg, Im Neuenheimer Feld 130.3, 69120 Heidelberg, Germany; 4 Institute of General Practice and Interprofessional Care, University Hospital Tübingen, Osianderstr. 5, 72076 Tübingen, Germany

**Keywords:** Spiritual care, Primary care, Loneliness, Medication, Old age, Home remedies, Naturopathic medicine, Alternative medicine, Self-efficacy, Self-care

## Abstract

**Background:**

Strategies to improve the care of elderly, multimorbid patients frequently focus on implementing evidence-based knowledge by structured assessments and standardization of care. In Germany, disease management programs (DMPs), for example, are run by general practitioners (GPs) for this purpose. While the importance of such measures is undeniable, there is a risk of ignoring other dimensions of care which are essential, especially for elderly patients: their spiritual needs and personal resources, loneliness and social integration, and self-care (i.e., the ability of patients to do something on their own except taking medications to increase their well-being). The aim of this study is to explore whether combining DMPs with interventions to address these dimensions is feasible and has any impact on relevant outcomes in elderly patients with polypharmacy.

**Methods:**

An explorative, cluster-randomized controlled trial with general practices as the unit of randomization will be conducted and accompanied by a process evaluation. Patients aged 70 years or older with at least three chronic conditions receiving at least three medications participating in at least one DMP will be included. The control group will receive DMP as usual. In the intervention group, GPs will conduct a spiritual needs assessment during the routinely planned DMP appointments and explore whether the patient has a need for more social contact or self-care. To enable GPs to react to such needs, several aids will be provided by the study: a) training of GPs in spiritual needs assessment and training of medical assistants in patient counseling regarding self-care and social activity; b) access to a summary of regional social offers for seniors; and c) information leaflets on nonpharmacological interventions (e.g., home remedies) to be applied by patients themselves to reduce frequent symptoms in old age. The primary outcome is health-related self-efficacy (using the Self-Efficacy for Managing Chronic Disease 6-Item Scale (SES-6G)). Secondary outcomes are general self-efficacy (using the General Self-Efficacy Scale (GSES)), physical and mental health (using the Short-Form Health Survey (SF-12)), patient activation (using the Patient Activation Measure (PAM)), medication adherence (using the Medication Adherence Report Scale (MARS)), beliefs in medicine (using the Beliefs About Medicines Questionnaire (BMQ)), satisfaction with GP care (using selected items of the European Project on Patient Evaluation of General Practice (EUROPEP)), social contacts (using the 6-item Lubben Social Network Scale (LSNS-6)), and loneliness (using the 11-item De-Jong-Gierveld Loneliness Scale (DJGS-11)). Interviews will be conducted to assess the mechanisms, feasibility, and acceptability of the interventions.

**Discussion:**

If the interventions prove to be effective and feasible, large-scale implementation should be sought and evaluated by a confirmatory design.

**Trial registration:**

German Clinical Trials Register (DRKS), DRKS00015696. Registered on 22 January 2019.

## Background

In most industrial countries the proportion of older people in the general population is constantly increasing due to lower birth rates and improved medical treatment options. In Europe, the mean life expectancy is currently 83.1 years for women and 77.5 years for men. About 20% of the European population is older than 65 years and this trend is rising since the fertility rate is 1.6 children per European woman, lower than necessary to keep the population stable [1]. Elderly people frequently suffer from multiple chronic diseases and are consequently treated with polypharmacy. Across Europe, 31% of older adults take five medications or more per day [2]. Multimorbid patients with polypharmacy have a higher risk for potentially avoidable hospitalizations and adverse drug reactions causing a substantial proportion of healthcare costs [3, 4]. Furthermore, they experience significantly lower quality of life than those with single diseases or taking fewer drugs [5].

Because of this, elderly patients with multimorbidity and polypharmacy have been receiving increasing attention from healthcare research and health policies. Interventions to improve their care frequently focus on medication safety, the prevention of delirium and falls, or the maintenance of mobility and pain control. Common strategies to achieve these goals are the provision of knowledge (e.g., by involving pharmacists into care), comprehensive geriatric assessments, or disease and case management [6, 7]. In Germany, disease management programs (DMPs) for individuals with diabetes, coronary heart disease and asthma/chronic obstructive pulmonary disease (COPD) are run by general practitioners (GPs). The core intervention of DMPs are regular appointments every 3–6 months during which a treatment protocol has to be completed.

Although the value of implementing standards and evidence-based knowledge into healthcare is undeniable, there is a risk of focusing healthcare for elderly patients too much on standardized measures overlooking other, more individual dimensions of care which also influence perceived quality of life. In a large German survey among elderly patients, self-efficacy was the most important determinant for well-being [8]. Self-efficacy can be defined as a person’s estimate of his or her own ability to succeed in reaching a specific goal, e.g., reducing symptoms or maintaining good health. Consequently, strengthening patients’ self-efficacy has the potential to substantially improve their quality of life. Furthermore, qualitative and quantitative research suggests that stronger self-efficacy is associated with better medication adherence [9–11].

**Fig. 1:**
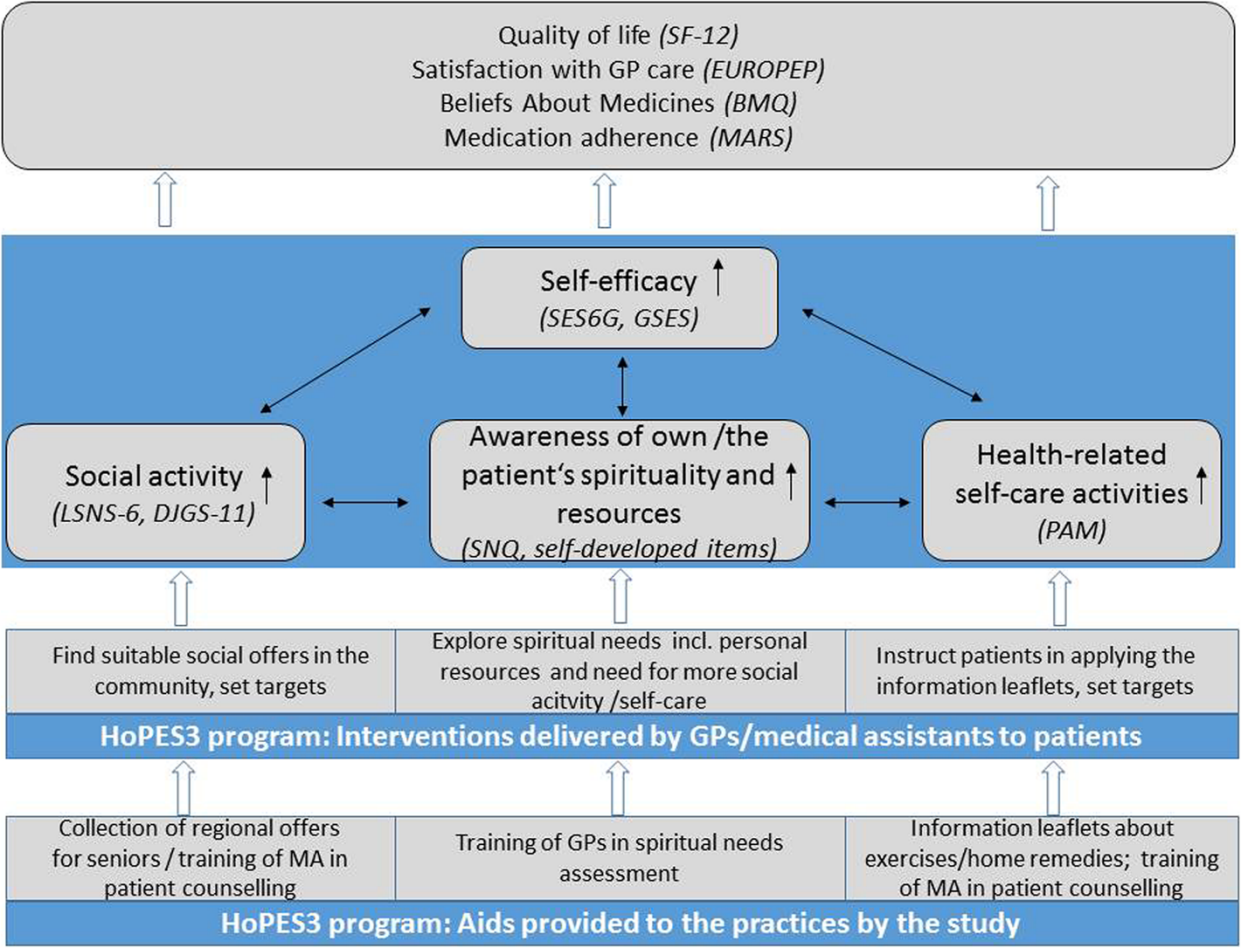
Logic model of the HoPES3 program. BMQ Beliefs About Medicines Questionnaire, DJGS-11 11-Item De-Jong-Gierveld Loneliness Scale, EUROPEP European Project on Patient Evaluation of General Practice, GP general practitioner, GSES General Self-Efficacy Scale, LSNS-6 6-Item Lubben Social Network Scale, MA medical Assistant, MARS Medication Adherence Report Scale, PAM Patient Activation Measure, SES6G Self-Efficacy for Managing Chronic Disease 6-Item Scale, SF-12 Short-Form Health Survey, SNQ Spiritual Needs Questionnaire

As Fig. 1 shows, the intervention evaluated in this study consists of three major components addressing three interlinked domains related to self-efficacy (spiritual needs, social activity, and self-care) on which we will elaborate below.

### Spiritual needs

To be able to strengthen patients’ self-efficacy it is necessary to be aware of their personal resources and their spirituality. The conceptual definitions of spirituality vary considerably [12]. In the context of healthcare, it is important to understand that spirituality is considered a dimension of the human being meaning that all humans have spiritual needs which differ in form and content. For this study, we define spiritual needs as everything which gives meaning to a person’s life and thus serves as a personal resource.

A representative survey among Germans revealed that the individual factors determining meaning of life change with age. While younger persons mentioned “friends”, “partnership”, or “work” as decisive factors, religious and nature experiences were most important to patients aged 70 or older [13]. A growing body of literature suggests connections between satisfaction of spiritual needs and mental and physical health [14]. It has been substantiated for a broad spectrum of conditions that a spiritual needs assessment reveals important information for the treatment of, for example, schizophrenia [15], chronic kidney disease [16], diabetes [17], or heart failure [18]. Several studies emphasize the role of spiritual needs for medication adherence. A cross-sectional study found that “spirituality, religiosity and personal beliefs were the only variables consistently associated with compliance to medication” and suggested that “adequately addressing these aspects on patient’s care may lead to an improvement in adherence patterns” [18]. Another cross-sectional study showed that adherent patients presented higher intrinsic religiosity [11]. Patients who were less trusting that a “powerful other” will take care of them were more likely to use unnecessary drugs [10]. An interview study with 51 patients with hypertension concluded that hopeless patients “adhere insufficiently or drop out completely, only improving their adherence if something happens that gives them hope or strength, or if they find a medical team that provides enough emotional support” [9].

Research shows that the majority of patients have a strong interest in discussing spirituality in medical encounters [19], and yet this aspect of care tends to be ignored, except in some special fields such as palliative care where spiritual care is being more and more implemented. While 85% of primary care physicians think they should be aware of patients’ spirituality, most would not initiate a talk about it except for dying patients [20]. For many physicians, spirituality is an area that makes them feel uncomfortable due to difficulties with spiritual language and worries about time, ethical boundaries, and reluctant reactions of the patient [14, 21, 22]. It has been suggested that acquiring basic skills in spiritual needs assessment should be part of medical training. Studies on spiritual care training for physicians draw positive conclusions about the effectiveness and acceptance of the training by patients and physicians [23, 24] and, in the United States, curriculums and objective structured clinical examinations (OSCEs) for spiritual care have already been established [25].

### Loneliness/social activity

The perceived meaningfulness of life is strongly linked to the degree to which a person maintains social relationships [26]. In a survey among elderly people living in German nursing homes, “feeling connected with family” was the second most important spiritual need [27]. Lacking self-efficacy, especially ‘spiritual health efficacy’ relating to one’s perceived ability to generate spiritually based faith and inner strength, proved to be a strong predictor for loneliness in old age [28, 29]. ‘Social loneliness’, which refers to the objective degree of social isolation or number of social contacts, should be distinguished from emotional loneliness, which refers to the subjective feeling of being alone and which might also be the symptom of a psychological disease [30].

Unfortunately, many elderly people are not satisfied with their social relationships. About one-fifth of the patients aged > 65 years in general practice feel often or occasionally lonely and 55% of them would like to get support to increase their social activity, but only 15% of them ever talked to their GP about these issues [31, 32]. GPs rarely ask patients about loneliness and, if so, they do it indirectly due to a feeling of powerlessness and inability to provide adequate support [33]. However, several systematic reviews show that interventions to reduce loneliness in elderly people can be effective [34–36]. Loneliness in elderly people is a risk factor for increased disease burden involving cognitive impairment [37], pain, depression, and fatigue [38], and abuse of benzodiazepines [39]. Furthermore, loneliness is associated with delayed hospital stays and increased costs [40, 41]. However, not all elderly patients suffer from being lonely. ‘Freedom’ and ‘independence’ are positive dimensions attributed to loneliness [42, 43]. For general practice it is important not to avoid discussions about loneliness and to identify those patients who need support to increase their social activity.

### Self-care

In this project we define self-care activities as “activities patients can perform on their own except taking medications to increase their well-being”. The positive correlation between self-care and self-efficacy are well described, for example, in studies among individuals with type 2 diabetes [44]. Based on these studies, it may be summarized that self-efficacy is a mediation variable for self-care behavior associated with positive health outcomes [45]. This is also expressed by the concept of ‘self-care self-efficacy’ which is defined as “one’s perceived ability to perform relevant self-care activities” and sometimes seen as a dimension of self-management [45]. According to Buck et al., patient’s confidence in their ability to perform self-care is a strong predictor of physical and emotional quality of life [46].

A large number of health programs have been developed to improve patients’ self-care ability. These are mostly disease-specific and often based on Orem’s nursing theory of self-care [45, 47–51]. This theory is based on the assumption that people have a natural ability for self-care, and nursing should focus on improving self-care behavior. This aspect of self-efficacy is also considered by DMPs. As part of the DMPs, all patients should participate in training sessions which focus on disease-specific knowledge and skills (e.g., correct use of inhaler devices for COPD, nutrition counseling for diabetes, and so on) [52].

In this study we aim to advise patients about self-care activities to enhance their self-care abilities for frequent symptoms in old age which are not alarming for a severe disease, e.g., knee pain in chronic osteoarthritis, chronic sleeping disorders, and so on. The self-care activities will focus on simple exercises (e.g., relaxation exercises, balance exercises) and on “home remedies”.

Home remedies or folk remedies are not clearly defined and, as discussed by Parisius et al. [53], a scientific definition is lacking. They could be described as “a traditional therapy often utilizing natural products, nutritional supplements or physical measures. Its effectiveness may be supported by familial, local or culturally accepted stories or rituals” [54]. We decided to include home remedies as an element in our intervention because they are easy to adopt, and elderly people are generally used to home remedies for self-care [55]. The application of home remedies requires theoretical knowledge and practical skills and, thus, optimally corresponds to the self-care concept. It is hypothesized that the application of home remedies has a positive effect on self-efficacy.

### Objectives

The aim of this exploratory study and the accompanying process evaluation is to assess the effectiveness and feasibility of interventions to strengthen patients’ self-efficacy by exploring their spiritual needs and personal resources and by addressing loneliness and self-care if needed.

## Methods/design

### Trial design

**Fig. 2:**
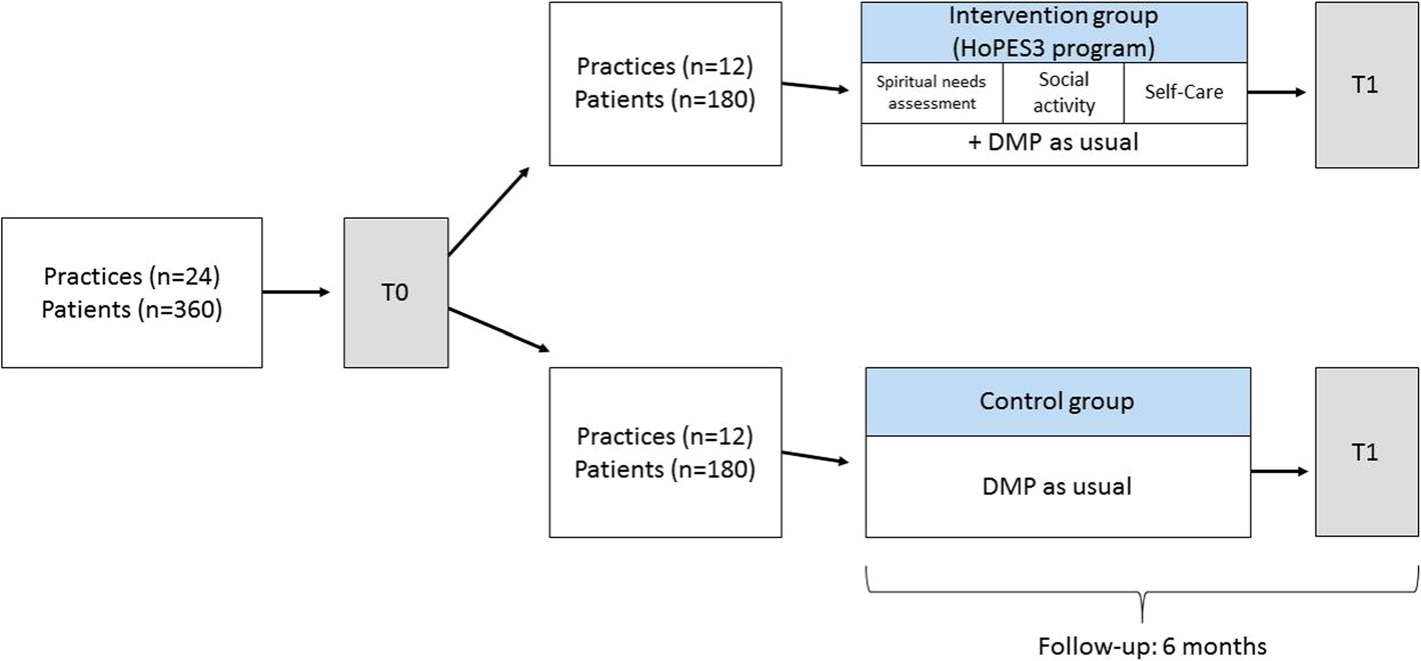
Trial design of the HoPES3 study. DMP disease management program

Figure 2 illustrates the trial design. An open, exploratory, cluster-randomized controlled trial with general practices as the unit of randomization and a follow-up time of 6 months will be conducted.

### Study setting

The interventions will be implemented in GP practices in the wider area around Heidelberg and Tübingen, two university cities in the federal state of Baden-Württemberg in southern Germany.

### Eligibility criteria

Inclusion criterion for GPs is participation in at least one DMP. Inclusion criteria for patients are: 1) age ≥ 70 years; 2) ≥ 3 chronic conditions defined by a previously published diagnosis list [56]; 3) ≥ 3 medications on a long-term basis; 4) participation in at least one DMP; and 5) ability to give informed consent, to take part in the spiritual needs assessment, and to complete the questionnaires (if necessary with assistance by a person not involved in the study) based on the GP’s personal assessment.

### Interventions

Two types of interventions should be distinguished.

#### Interventions delivered by GPs and medical assistants to patients

Patients in the intervention group will receive one prolonged counseling session as part of the regular appointment for the DMP during which their GP explores their personal resources and spiritual needs, including the need for more social contact and self-care activities. Based on previous research, we assume that the spiritual needs assessment will take 10–15 min [23]. Trained medical assistants will inform all patients about social activities for seniors in the community and about nonpharmacological treatment alternatives and, if appropriate, set targets with the patients (another 10–15 min). Patients in the intervention group will receive a diary and be asked to document when and how they conduct social or self-care activities.

#### Aids provided to the GP practices by the study

Patients, GPs, and medical assistants in the intervention group will have access to a web portal developed by the study team with a collection of social activities for seniors in the region near the respective practice. Comprehensive search options (radius search, thematic filters, map screens) will facilitate the identification of suitable offers. Additionally, the social activities will be made available as a brochure in print form. Furthermore, information sheets developed by the study team will be provided to the practices and patients in the intervention group in paper-based and digital forms describing self-care activities (simple exercises and home remedies) to reduce frequent symptoms in old age which are not alarming for a severe disease. GPs and medical assistants will participate in a 4- to 5-h workshop conceptualized and led by the authors of this protocol. For GPs, the workshop will comprise an introduction to the definition of spirituality, the concept of spiritual care, and a previously evaluated conversation technique (SPIR [23]), as well as self-reflection of their own spirituality and practical exercises on conducting a spiritual needs assessment based on videos of real patients and role plays with trained actors. The training of medical assistants will focus on patient counseling regarding self-care and social activity and on the handling of the web portal and the info sheets. Possible barriers and facilitators for implementing the HoPES3 program into practice will be discussed.

### Control

In the control practices, DMPs will be conducted as usual. Although DMPs are defined by a standardized treatment form to be completed during each patient contact, the way DMPs are organized in GP practices varies substantially. To get a better idea of usual care in our study, we will ask all participating GPs and medical assistants to describe how they usually organize DMPs in their practice.

### Outcomes

The primary and secondary outcomes of this study will be based on primary data collected in written, paper-based surveys. The rationale for choosing the various outcomes is shown schematically in Fig. 1. The primary outcome is patients’ self-efficacy measured by the Self-Efficacy for Managing Chronic Disease 6-Item Scale (SES6G). The German version of the SES6G showed good construct validity and high internal consistency [57]. In addition to health-related self-efficacy, general self-efficacy will be measured by the General Self-Efficacy Scale (GSES) [58].

Secondary outcomes were chosen to assess the effects of each of the single intervention components and of the entire HoPES3 Program. To assess the patients’ social activity, the short, six-item version of the Lubben Social Network Scale (LSNS-6) [59] and the 11-item De-Jong-Gierveld Loneliness Scale (DJGS-11) [30] will be used. The LSNS-6 determines the number of available contact persons (i.e., the degree of social isolation) and the DJGS-11 measures the perceived social and emotional loneliness. Patients’ self-care abilities will be estimated by the Patient Activation Measure (PAM) [60]. Awareness of own spirituality or the patients’ spirituality, respectively, will be assessed by self-developed items.

We hypothesize that the HoPES3 program will improve patient quality of life as assessed by the Short Form Health Survey (SF-12) [61], and satisfaction with GP care and patient-centered communication will be assessed by selected items of the European Project on Patient Evaluation of General Practice (EUROPEP) questionnaire [62]. To explore whether HoPES3 has any influence on the use of medications, the Medication Adherence Report Scale (MARS) [11] and the general part of Beliefs About Medicines Questionnaire (BMQ) [63] will be used. At the level of GPs and medical assistants, work satisfaction (using the Warr-Cook-Wall-Scale) [64], competence in providing spiritual care (using the Spiritual Care Competence Questionnaire) [65], or in advising self-care activities and awareness of own spirituality (using self-developed items) will be assessed.

### Other data

Besides outcome measures, descriptive data and process data will be collected. We will use the Spiritual Needs Questionnaire [66] and self-developed items to describe the type and intensity of the spiritual needs of the sample and whether their spirituality is a source of strength or rather a risk factor to them. Furthermore, we will collect printouts of the diagnosis lists and medication lists saved in the patient records of the participating practices. We will ask GPs, medical assistants, and patients in the intervention group to assess the usefulness of and strain caused by the interventions within 2 weeks after the spiritual needs assessment via a short self-developed questionnaire. A diary will be given to patients in the intervention group asking them to document how often and what type of social and self-care activities they apply. The log file of the web portal will be analyzed to determine which functions were used and how often.

After the intervention period terminates, interviews with GPs, medical assistants, and patients in the intervention group will be conducted, audiotaped, and transcribed for the purpose of a comprehensive process evaluation. Research questions of the process evaluation are:

- Intervention fidelity
  - Were the interventions delivered as specified in the protocol?
  - How is the acceptance of the interventions by GPs, medical assistants, and patients?
  - How feasible are the interventions for use in daily practice?
  - What are potential barriers and solutions for delivering the interventions as intended?
- Intervention mechanisms
  - What is the perceived benefit of the interventions for patients and healthcare professionals? What are potential harms?
  - Which subgroups of patients profit from the intervention, and which do not?
  - How did the interventions influence the patient–physician relationship?
  - How did the interventions influence medical treatment, especially medication adherence and use of unnecessary drugs?
- Intervention costs
  - How big is the effort for performing the interventions?

### Recruitment and participant timeline

**Fig. 3:**
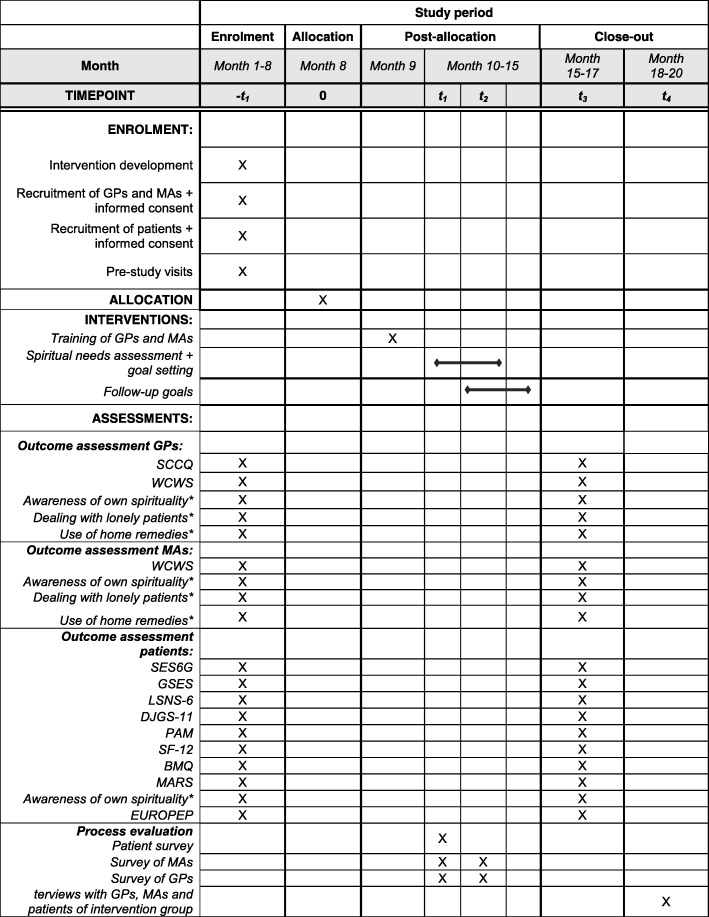
Time schedule of the HoPES3 study (following the SPIRIT recommendations). BMQ Beliefs About Medicines Questionnaire, DJGS-11 11-Item De-Jong-Gierveld Loneliness Scale, EUROPEP European Project on Patient Evaluation of General Practice, GP general practitioner, GSES General Self-Efficacy Scale, LSNS-6 6-Item Lubben Social Network Scale, MA medical Assistant, MARS Medication Adherence Report Scale, PAM Patient Activation Measure, SES6G Self-Efficacy for Managing Chronic Disease 6-Item Scale, SCCQ Spiritual Care Competence Questionnaire, SF-12 Short-Form Health Survey, WCWS Warr-Cook-Wall Scale

The time schedule of the study is depicted in Fig. 3. GPs offering DMPs will be recruited in the wider area of Heidelberg and Tübingen. For this purpose, the study information will be sent to the practices of already established research networks of the University Hospitals Heidelberg and Tübingen. Participating GPs will identify all patients meeting the eligibility criteria using the filter options of their practice software. GPs will be asked to estimate which patients would profit most from the HoPES3 program and to include 15 of these patients into the study. The reasons for selecting or excluding patients as well as for refusing to participate will be documented and explored in the process evaluation. All participants will give written informed consent. In case of patients, the informed consent forms will remain in the practices.

### Management, collection, and monitoring of data

All data will be managed at the study center located at the University Hospital Heidelberg, Department of General Practice and Health Services Research. The study center will receive patient data only in pseudonymized form and will not have direct contact with patients (except for interviews with patients who gave their permission to be contacted by the study team). Only authorized staff members are able to enter or edit data. For specific research questions, parts of the data will be made available to partners of the project (University Hospitals Munich and Tübingen) in pseudonymous form after the trial biometrician has finalized the statistical report. The partners guarantee that only the analyzing researchers will have access to the data. It is also planned to make trial data on which scientific publications are based and all the primary data publicly available for re- and meta-analyses after the trial has been completed.

A study nurse will monitor the data entry during the entire study period. Furthermore, the study team will visit all participating practices before randomization to explain organizational issues related to the study. A Data Safety and Monitoring Board (DSMB) consisting of a GP, a statistician, and an expert in spiritual care will be regularly informed about the course of the trial and all safety issues, and asked for advice whether to continue, modify, or stop the trial.

### Sample size

The sample size was calculated using the primary endpoint, the SES6G at T1. With *n* = 22 practices (11 per group) and *n* = 264 (132 per group) patients available for analysis, it will be possible to detect a clinically relevant mean difference of δ = 1 point at T1 between the two treatment groups for the mean SES6G score at a two-sided α of 0.05 with a power of 1 – β = 0.8, assuming a standard deviation of σ = 2.2 based on the results of Lorig et al. [67], a relatively high intraclass correlation coefficient [68] of 0.05, and a cluster size of *m* = 12 (the calculation was done using the function n4means from the R package CRTSize). It is expected that, adjusting for the SES6G score at T0, gender, age, number of comorbidities, and number of medications in the linear mixed model used for statistical analysis will lead to less unexplained variance and thus to an increase in power. Taking drop-out rates of 7.5% at the practice level and 20% at the patient level, respectively, into account, *n* = 24 practices with *n* = 360 patients will be enrolled into the trial. The potential problem of missing values for drop outs will be partly resolved in the primary analysis by application of the pre-defined imputation strategy.

### Statistical methods

The primary objective of this study is to determine the effectiveness of a complex intervention compared with usual care. The primary efficacy endpoint is the SES6G score at T1. The study objective is statistically formulated as a test of the null hypothesis H_0_: μ_I_ = μ_C_ (the mean SES6G score at T1 in the intervention and the control group are equal) against its alternative H_1_: μ_I_ ≠ μ_C_ at a significance level of α = 0.05 (two-sided).

Because of the cluster randomization, the primary efficacy analysis will use a multilevel regression approach with patients at level one and practices at level two. The primary efficacy analysis will be performed by fitting a linear mixed model including the SES6G score at T1 as the dependent variable, treatment group and gender as fixed factors, and SES6G score at T0, age, number of comorbidities, and number of medications as fixed covariates, and practice as a random factor to account for the two-level data structure (patients nested within practices). The results will be presented as the mean between-group difference in SES6G at T1 with the corresponding 95% confidence interval. The associated Cohen’s effect size *d* will be calculated. In addition, the practice-related intracluster correlation coefficient (ICC) will be estimated. The primary analysis will be performed adhering to the intention-to-treat principle. An additional sensitivity analysis will be conducted on the per-protocol analysis set. Missing data for the primary outcome variable will be replaced using multiple imputation [69] which takes the covariates of treatment group, gender, age, number of comorbidities, number of medications, and practice into account by application of the fully conditional specification method [70]. Sensitivity analyses will be performed by applying alternative methods dealing with missing data such as, for example, complete case analysis and best/worst case imputation.

The statistical analyses of the secondary endpoints will use the same multilevel approach as the primary analysis. All statistical tests will be two-sided at the significance level of α = 0.05. Because no adjustments for multiple endpoints are planned, findings will be interpreted with caution in view of the number of statistical tests undertaken. Only the result of the primary efficacy analysis will be interpreted in a confirmatory manner. Confirmatory subgroup analyses are not planned. No interim analysis with regard to efficacy will be done. All analyses will be performed using SAS version 9.4 or higher.

### Assignment of interventions

As Fig. 2 shows, 360 patients and 24 practices will be recruited (half in the area of Heidelberg, half in the area of Tübingen) and randomized to either intervention or control group using block randomization stratified by region (Heidelberg/Tübingen) to ensure an equal number of intervention and control practices per region. The randomization list will be created by the trial statistician. Since the intervention involves training of GPs and informed consent of patients, blinding of participants is not possible. However, acknowledged measures to reduce bias such as computerized randomization and blinding of outcome assessment will be undertaken.

### Harms

The pharmacological treatment of the patients is not altered by the study, and therefore we do not expect severe risks to health. However, it is possible that the spiritual needs assessment causes psychological strain for the participating patients and/or GPs. Although the strain caused by the conversation technique (SPIR) that will be applied in this study was considered low in previous studies, we will ask patients and GPs to evaluate the strain within 2 weeks after each assessment by means of a questionnaire. The responses of each patient and those aggregated at a practice level will be discussed with the DSMB.

## Discussion

To our knowledge, HoPES3 is the first study examining interventions to address spiritual needs, social activity, and self-care in elderly patients in German general practice.

Since little is known about the effectiveness of such interventions, HoPES3 is designed as an explorative trial. This means we have chosen a range of secondary outcomes beside the primary outcome to assess the possible effects of the program. If the program proves to be effective and feasible, large-scale implementation should be sought and evaluated by confirmatory research. This could be done, for instance, by integrating the training concept into the university curricula for medical students or into continuous education of GPs or by collaborating with health insurance companies or local state authorities.

In German language the term spirituality is frequently associated with esotericism or religion. We are aware that some GPs and maybe also patients might have a negative attitude towards this topic. Therefore, we will explain in the invitation letter and study information how spirituality is defined in our project and that a spiritual needs assessment is a way of resource-oriented communication which has the potential to reveal important information for the treatment of the patient. However, we cannot exclude that there will be a selection bias in favor of those GPs and patients who are already interested in the topic and assess this aspect during the baseline assessment.

In this study, GPs will select those patients who, in their opinion, will profit most from the intervention. While this approach may be criticized in clinical research and efficacy trials because it limits the generalizability of the findings, it is justifiable in health services research which focusses on the effectiveness of interventions under real-life conditions. It is common in general practice that GPs decide which treatment or which conversation technique is appropriate for which patient. Therefore, we believe that the selection of patients by GPs is a realistic approach which would also be applied in case the HoPES3 program is implemented into routine care. The reasons for selecting or excluding patients will be examined and will reveal important information for the adaption and dissemination of the program.

## Data Availability

Not applicable.

## Abbreviations

BMQ: Beliefs About Medicines Questionnaire
DJGS-11: 11-Item De-Jong-Gierveld Loneliness Scale
DMP: Disease management program
DRKS: German Registry for Clinical Trials
EUROPEP: European Project on Patient Evaluation of General Practice
GP: General practitioner
GSES: General Self-Efficacy Scale
LSNS-6: 6-Item Lubben Social Network Scale
MARS: Medication Adherence Report Scale
PAM: Patient Activation Measure
SES6G: Self-Efficacy for Managing Chronic Disease 6-Item Scale
SF-12: Short-Form Health Survey
